# Relationship between benzodiazepine prescription, aggressive behavior, and behavioral disinhibition: a retrospective study in a Swiss prison

**DOI:** 10.1186/s12954-021-00504-5

**Published:** 2021-05-20

**Authors:** Stphanie Baggio, Vladan Starcevic, Patrick Heller, Karen Brndle, Irina Franke, Andreas Schneeberger, Anna Buadze, Alex Gamma, Roman Schleifer, Laurent Gtaz, Hans Wolff, Michael Liebrenz

**Affiliations:** 1 Division of Prison Health, Geneva University Hospitals and University of Geneva, Chemin du Petit Bel Air 2, 1226 Thnex, Geneva, Switzerland; 2 Office of Corrections, Department of Justice and Home Affairs of the Canton of Zurich, Zurich, Switzerland; 3 Faculty of Medicine and Health, Sydney Medical School, Nepean Clinical School, Discipline of Psychiatry, University of Sydney, Sydney, NSW Australia; 4 Adult Psychiatry Division, Department of Mental Health and Psychiatry, Geneva University Hospitals, Geneva, Switzerland; 5 Department of Forensic Psychiatry, Institute of Forensic Medicine, University of Bern, Bern, Switzerland; 6 Psychiatrische Dienste Graubnden (PDGR), Chur, Switzerland; 7 Department of Psychiatry and Behavioral Sciences, Montefiore Medical Center, Albert Einstein College of Medicine, New York, USA; 8 Department of Psychiatry, Psychotherapy and Psychosomatics, Psychiatric Hospital, University of Zurich, Zurich, Switzerland

**Keywords:** Benzodiazepine, Drug prescription, Health care, Prison

## Abstract

**Background:**

Benzodiazepines are commonly prescribed in prisons amidst the controversies surrounding their potential role in causing behavioral disinhibition and aggressive behavior and their association with use and trafficking of illicit and addictive substances. The present study aimed to (1) ascertain the relationship between benzodiazepine prescription (including their dosage and duration of use) and aggressive behavior and behavioral disinhibition in prison and (2) investigate whether there was an association between benzodiazepine prescription, (including their dosage and duration of use) and using and trafficking illicit and addictive substances during imprisonment.

**Methods:**

Data were extracted from the electronic database of an open Swiss prison (*n*=1206, 1379 measures) over a 5-year period (20102015). Measures included benzodiazepine prescription, duration of benzodiazepine use and mean dosage, and punishable behaviors (physical and verbal aggression, disinhibited but not directly aggressive behaviors, property damage or theft, substance-related offenses, and rule transgression). We assessed the relationship between benzodiazepine prescription and punishable behaviors after propensity score matching. Logistic regressions were also used to test the relationship of benzodiazepine use duration and dosage with punishable behaviors among participants who received benzodiazepines.

**Results:**

After propensity score matching, benzodiazepine prescription was not significantly associated with any punishable behavior. Among detained persons who took benzodiazepines, there was no significant association of dosage and duration of use with offenses involving illicit or addictive substance use or trafficking.

**Conclusions:**

Our study did not empirically support the occurrence of increased aggressive or disinhibited behaviors or increased risk of substance abuse in detained persons who received benzodiazepines in prison. This suggests a need to reconsider restrictions in prescribing benzodiazepines in the prison setting.

**Supplementary Information:**

The online version contains supplementary material available at 10.1186/s12954-021-00504-5.

## Introduction

Benzodiazepines (BZD) are one of the most widely prescribed drugs in Western, Educated, Industrialized, Rich, and Democratic (WEIRD) countries [1,5]. They are mainly prescribed to treat sleep disorders, anxiety disorders, epilepsy, and withdrawal from certain substances. Their use has been widely debated. On the one hand, BZD act quickly, are very useful in acute settings, and are generally effective. On the other hand, they have been associated with various adverse effects, including sedation, psychomotor and cognitive impairment, falls and fractures in the elderly, and dependence [6, 7]. In addition, individuals with substance use disorders frequently abuse BZD, which is harmful, especially when various illicit drugs and opioids are combined with BZD [8]. In view of these problems, most treatment guidelines do not recommend BZD as first-line treatment for anxiety and related disorders and suggest that they should only be used short-term [9,11]. In contrast, several experts have recently pointed out that such recommendations are based on little empirical evidence and that these guidelines do not adequately reflect the risk-to-benefit ratio when using BZD [3, 4, 12,15].

If use of BZD is controversial in the general population, the situation is even more complex in prisons, where they are commonly prescribed [16, 17]. First, substance use disorders are more common among detained persons than in the general population [18], and smuggling and trafficking of drugs are frequent in prisons around the world [19]. Considering the likelihood of BZD abuse in the context of substance use disorders, it has been suggested that BZD should be entirely avoided or minimally prescribed in prisons [16, 20,22]. However, this is problematic, because prisoners would be deprived of a valid therapeutic option [2, 23].

Another crucial issue associated with prescribing BZD in prisons pertains to an increased risk of behavioral disinhibition, resulting in aggressive behavior [1, 24]. There is a dearth of research on this topic [25], and some studies suggest that the link between BZD use and heightened aggression may only apply to short-acting BZD [26]. Furthermore, violent crime was associated with unusually high doses of BZD [27], whereas there was no increase in impulsive behavior with therapeutic doses of BZD [28]. In a recent study, Albrecht et al. [29] concluded that high BZD doses were not sufficient to increase the risk of violence. These disparate findings make it difficult to understand the role of BZD as a treatment option in the prison setting.

In view of the aforementioned issues and controversies, the present study, conducted in a sample of Swiss detained persons, had two main aims: (1) to ascertain the relationship between BZD prescription and aggressive behavior and behavioral disinhibition in prison; we also aimed to assess the potential effects of the dosage of BZD and duration of their use on aggression and behavioral inhibition during imprisonment; and (2) to investigate whether BZD prescription was associated with using and trafficking illicit and addictive substances.

## Materials and methods

### Study design and participants

This retrospective cohort study was based on the data on 1206 persons detained in Realta prison, KantonGraubnden, Switzerland. This is an open prison (capacity of 120) for sentenced males who work outside the prison and have up to 36h of leave per week. Data for the 20102015 period were extracted from the electronic prison database (Gina, Ultrasoft AG) on 1379 measures. (Some detained persons were incarcerated multiple times.)

Administration of all medications is strictly regulated in the Swiss correctional facilities and follows directions from the Swiss Centre of Expertise in Prison and Probation, which supports the Swiss Conference of Cantonal Justice and Police Directors. Medications under a controlled prescribing regimen (e.g., BZD) are prescribed by the prison medical team and administered in a strictly specified manner: Tablets are crushed by nurses, dissolved in water, and consumed in the health service office under nurses supervision. PRN administration of BZDs was not allowed and not included in the analyses. Detained persons are at not allowed to carry any prescription medication outside the health service office. Approval for conducting the study was received from the Cantonal Research Ethics Committee of Bern (no. 2016-01539).

### Variables

1. *Socio-demographics* These include age and region of origin.
2. *Incarceration and offense variables* We recorded the length of incarceration (based on the dates of admission and discharge) and offenses leading to imprisonment: violence-related offenses (assaults, sexual crime, other kinds of violence); property-related offenses (theft, robbery, other property offenses); substance-related offenses (violation of drug laws); and other offenses (arson, justice obstruction, manslaughter, trafficking, violation of weapon laws).
3. *BZD prescription* We recorded whether or not detained persons were prescribed BZD during imprisonment. For those who received BZD, duration (in days) of use and dosage (mean dosage expressed as mg/day and converted into diazepam mg equivalents) of BZD were collected. The list of BZD and diazepam mg equivalent conversion guidelines are provided in Additional file 1: Table S1.
4. *Other prescribed medications* We recorded whether detained persons were prescribed other psychotropic medications (i.e., antidepressants, antipsychotics, methylphenidate, mood stabilizers, or opioid agonists [including heroin used for therapeutic purposes]) and medications for any somatic (non-psychiatric) condition.
5. *Punishable behaviors* Data on the type and frequency of detained persons punishable behaviors were collected. These were classified into five categories: (1) physical and verbal aggression (assaults and threats made against others), (2) disinhibited, but not directly aggressive behaviors (e.g., slamming doors, swearing), (3) property damage or theft, (4) substance-related offenses (alcohol or illicit drug use or trafficking), and (5) rule transgression (e.g., smoking when not allowed, returning from leave late). We made a binary qualification (presence/absence) for each category.

### Statistical analyses

We first computed descriptive statistics for all variables. Then, we tested whether punishable behaviors were associated with BZD prescription.

In the first set of analyses, we used a propensity score matching to minimize the effect of confounding factors and make it possible to compare individuals who received BZD with those who did not receive them. This method is used to estimate the effect of a treatment when relying on observational data and to address the fact that assignment to a treatment is not random. The propensity score matching aims to mimic randomization by matching the treated and untreated groups on a set of predetermined covariables. The propensity score was first derived using group assignment (BZD/no BZD prescription) predicted by factors that might influence group assignment: age, region of origin (Switzerland/outside of Switzerland), length of incarceration, type of offense, use of any other psychotropic medication, and use of any medication for somatic diseases. Outcome variables (punishable behaviors) were not included in this first step of the analysis. The propensity score was estimated using a probit regression. Each participant received a continuous propensity score, which constituted the conditional probability of having a group assignment (BZD/no BZD prescription) with given covariates. We then used the propensity score to match participants in each group (BZD/no BZD) with the nearest neighbor matching (fill Mahalanobis), allowing multiple neighbors in case of identical propensity scores. Therefore, participants with the same propensity scores were matched and considered comparable on covariates used to derive the propensity score. Again, the matching was done without consideration of the outcome variables. Finally, the association between matched groups and punishable behaviors was tested, computing the average treatment effect on the treated (ATT, here the effect of having BZD/no BZD prescription) for each punishable behavior used as outcome (sanctions related to physical and verbal aggression, disinhibited, but not directly aggressive behaviors, property damage or theft, substance-related offenses, and rule transgression). Crude and matched associations (before/after propensity score matching) are reported. The balancing properties of the propensity score were satisfied.

In the second set of analyses that did not involve propensity score matching, we focused on detained persons who received BZD. We computed five logistic regressions, using as predictors mean dosage and duration of BZD prescription and the same outcomes as the first set of analyses. For all these models, we controlled for age, region of origin, length of incarceration, type of offenses, use of any other psychotropic medication, and use of any medication for somatic diseases.

For the propensity score analysis, we computed sensitivity analyses using other methods to match groups (covariate adjustment, inverse probability weighting, and stratification). For the second set of analyses, we performed sensitivity analyses by using the maximum BZD dosage instead of the mean dosage. We also conducted logistic regressions using mean dosage and duration of BZD use coded as zero for detained persons without BZD prescription. We also controlled for the effect of short- versus long-acting BZD. Finally, to take into account data clustering, we ran (1) mixed-effect models on the whole sample and on the subsample of participants with BZD prescription, to see whether there was a difference when considering that some measures were nested into participants; and (2) the same analyses (including propensity score matching) using the first incarceration of each participant. In all cases, the findings were similar to those reported in the Results section.

Analyses were performed with Stata 15 (propensity score estimation: *pscore* with no imposition of common support, propensity matching: *psmatch2* with option ties).

## Results

Descriptive statistics are reported in Table 1. The mean age of detained persons was 33.110.4years. About a third (35.4%) came from Africa and another third (31.4%) from Western Europe. The mean duration of incarceration was 125.1days.

**Table 1:** Descriptive statistics and bivariate analyses for socio-demographics, incarceration and offense variables, other prescribed medications, and punishable behaviors

Variables	Whole sample (*n*=1379)	BZD prescription	
		Yes (*n*=293)	No (*n*=1,086)
Socio-demographics			
Age^a^	33.1 (10.4)	35.4 (9.8)	32.5 (10.5)
Region of origin^b,c^			
Asia	1.2 (17)	0.7 (2)	1.4 (15)
Eastern Europe/Balkans	11.6 (160)	8.9 (26)	12.3 (134)
Eastern, Central, and South Africa	4.1 (57)	1.4 (4)	4.9 (53)
Latin America	1.7 (23)	1.7 (5)	1.7 (18)
Middle East	6.7 (93)	4.8 (14)	7.3 (79)
North Africa	19.2 (265)	21.8 (64)	18.5 (201)
Switzerland	24.4 (337)	37.2 (109)	21.0 (228)
Western Africa	12.0 (166)	3.1 (9)	14.5 (157)
Western Europe	7.0 (96)	6.8 (20)	7.0 (76)
Unknown/unverified	12.0 (165)	13.7 (40)	11.5 (125)
Prison variables			
Length of incarceration (days)^a^	125.1 (177.4)	157.3 (171.6)	116.4 (178.1)
Type of offense^c^			
Violence^b^	13.1 (180)	15.4 (45)	12.4 (135)
Property^b^	38.7 (533)	54.6 (160)	34.4 (373)
Substance^b^	19.4 (267)	25.3 (74)	17.8 (193)
Other^b^	60.0 (827)	51.2 (150)	62.3 (677)
Medical information			
Benzodiazepine			
Prescription^b^	21.3 (293)		
Duration (no. of days)^a,d^	91.5 (7.0)		
Mean daily dosage (mg/Diazepam equivalent)^a,d^	24.0 (25.0)		
Prescription of other psychotropic medications			
Any^b^	25.0 (345)	68.3 (200)	13.4 (145)
Antidepressant^b^	11.8 (163)	34.5 (101)	5.7 (62)
Antipsychotic^b^	15.7 (217)	43.3 (127)	8.3 (90)
Methylphenidate^b^	2.9 (27)	7.2 (21)	0.6 (6)
Mood stabilizers^b^	1.5 (21)	3.8 (11)	0.9 (10)
Opioid antagonist^b^	4.9 (68)	16.0 (47)	1.9 (21)
Other^b^	0.9 (13)	3.1 (9)	0.4 (4)
Any medication for somatic disease^b^	32.4 (447)	56.3 (165)	26.0 (282)
Punishable behaviors (outcomes)			
Physical and verbal aggression^b^	5.9 (81)	8.5 (25)	5.2 (56)
Disinhibited but not directly aggressive behavior^b^	4.7 (65)	7.9 (23)	3.9 (42)
Property damage or theft^b^	2.8 (38)	3.8 (11)	2.5 (27)
Substance-related offenses^b^	11.7 (161)	19.1 (56)	9.7 (105)
Rule transgression^b^	23.4 (323)	31.7 (93)	21.2 (230)

^a^Means and standard deviations

^b^Percentages and *n*

^c^There were 165 missing values for the region of origin (12.0%) and 40 missing values for type of offenses (2.9%)

^d^Reported for participants with BZD prescription (*n*=290)

A total of 293 (21.3%) detained persons were prescribed BZD during their incarceration (mean duration of BZD use=91.5days, mean dosage=24.0mg/day diazepam equivalents).

The most common type of punishable behavior was rule transgression (323 instances or 23.4% of the total sample), followed by substance-related offenses (161; 11.7%), physical and verbal aggression (81; 5.9%), disinhibited, but not directly aggressive behaviors (65; 4.7%), and property damage or theft (38; 2.8%).

In the analyses of the unmatched sample, detained persons with and without BZD prescription were significantly different in terms of the factors included in the propensity score and outcomes (left panel of Table 2). In the matched sample, there was no significant difference on any factor included in the propensity score (right panel of Table 2). Thus, BZD prescription was not significantly associated with any kind of punishable behavior.

**Table 2:** Associations of factors and outcomes with BZD prescription in a sample with and without propensity score matching

	Sample without propensity score matching			Sample with propensity score matching		
	BZD	No BZD	*p*	BZD	No BZD	*p*
	*n*=293	*n*=1086		*n*=253	*n*=964	
Variables included in the propensity score						
Age^a^	35.59	32.47	<.001	35.59	35.59	.643
Region of origin (ref. Switzerland)^b^	0.43	0.24	<.001	0.43	0.46	.532
Length of incarceration (no. of days)^a^	158.30	115.37	<.001	158.30	134.51	.122
Prescription of other psychotropic medications^b^	0.67	0.14	<.001	0.67	0.67	.925
Prescription of medication for somatic disease^b^	0.55	0.25	<.001	0.55	0.54	.789
Offence: violence^b^	0.15	0.13	.231	0.15	0.14	.616
Offence: property^b^	0.53	0.33	<.001	0.53	0.56	.423
Offence: substance^b^	0.25	0.18	.018	0.25	0.22	.464
Offence: other^b^	0.52	0.62	.002	0.52	0.53	.722
Punishable behaviors (outcomes)^b^						
Physical and verbal aggression	0.08	0.05	.084	0.08	0.04	.194
Disinhibited but not directly aggressive behavior	0.17	0.10	.001	0.17	0.13	.390
Property damage or theft	0.08	0.04	.001	0.08	0.07	.576
Substance-related offenses	0.03	0.03	.617	0.03	0.02	.390
Rule transgression	0.30	0.21	.003	0.30	0.24	.239

*BZD* benzodiazepine

^a^Means are reported

^b^Proportions are reported

With regard to detained persons who were prescribed BZD, the mean dosage of BZD was not associated with any kind of punishable behavior (Table 3). Duration of BZD prescription was significantly associated only with disinhibited, but not directly aggressive behaviors (*p*=.011).

**Table 3:** Association between BZD dosage and duration of use and punishable behaviors (*n*=290)

DV	BZD mean dosage (IV1)		BZD duration (IV2)	
	coef.	*p* value	coef.	*p* value
Physical and verbal aggression	0.001	.888	0.001	.847
Disinhibited but not directly aggressive behavior	0.021	.110	0.010	.011
Property damage or theft	0.010	.399	0.005	.343
Substance-related offenses	0.003	.611	0.002	.291
Rule transgression	0.008	.171	0.001	.522

Logistic models were adjusted for age, region of origin (Switzerland/not Switzerland), length of incarceration, type of offenses, prescription of any other psychotropic medication, and prescription of any medication for somatic diseases

*BZD* benzodiazepine, *DV* dependent variable, *IV* independent variable

## Discussion

The main aim of the present study was to investigate the effects of BZD prescription on aggressive behaviors and behavioral disinhibition in a Swiss prison. When controlling for potentially confounding variables, we did not find any association between BZD prescription and punishable behaviors. This suggests that control variables might have captured a previous tendency towards aggressive behavior (reflected by the type of offenses) and psychiatric disorders associated with aggressiveness or disinhibited behaviors (reflected by the prescription of other psychotropic medications). This finding is in line with studies showing that therapeutic doses of BZD are not associated with heightened aggressive behavior [28, 29], but it is in contrast to other research that reports an association between use of BZD and aggressive behavior [1, 24]. Importantly, we also found no association between the dosage of BZD and duration of their use and almost all kinds of punishable behaviors among detained persons who received BZD.

The only significant association we identified was between use of BZD for longer periods of time (assessed as a continuous variable, via number of days of prescription) and presence of disinhibited, but not directly aggressive behaviors. However, the effect size of this finding was very small: With an increase in the duration of BZD use by 1day, detained persons were 1.01 time more likely to exhibit disinhibited, but not directly aggressive behaviors.

The second aim of the study was to investigate whether BZD prescription was associated with using and trafficking illicit or addictive substances during imprisonment. We found that detained persons taking BZD were not more likely to commit offenses involving illicit or addictive substance use or trafficking. This did not change when we examined the dosage and duration of BZD use. This finding has important implications because prescribing BZD in prisons is often avoided on the grounds of their presumed greater abuse potential [20, 21].

Our study has also revealed other important findings about the use of BZD in the prison setting. The proportion of detained persons who were prescribed BZD (21.3%) was by no means negligible. Moreover, when compared with detained persons who were not prescribed BZD, those using BZD were also prescribed significantly more often all other classes of psychotropic agents and medications for general medical conditions. It indicates their high need for adequate healthcare.

Following the principle of the equality of care, prison populations should benefit from effective and evidence-based treatments that are available in the community. Our study suggests that BZD in the prison setting should not be routinely denied and that they should be available under a high standard of supervised medication delivery [2, 23]. The same applies to other psychotropic medications, as well as psychological interventions for mental disorders. It is important to acknowledge that there are very few alternatives to psychotropic medications in the prison system [30] and that treatment of many mental health issues should not only rely on medications such as BZD.

### Limitations

This study has a number of limitations. First, it was based on associations and was conducted retrospectively, which precludes us from making inferences about any causal relationship. Second, punishable behaviors recorded by the prison administration were the only indicator of aggressive and disinhibited behavior, and we had no access to any information about punishable behaviors that were concealed or undetected. Future studies should be conducted prospectively and use instruments for assessing irritability, anger, behavioral disinhibition, and aggressiveness. Third, our findings are based on a sample of male detained persons in a Swiss setting and it is uncertain to what extent they can be applied to female detained persons in another country. Fourth, there were no data on psychiatric diagnoses and specific indications for prescribing BZD, which might have been helpful to better understand and contextualize the risk of aggressive behavior and disinhibition. Fifth, prescribing any medication does not necessarily mean that it will be taken as prescribed. However, BZD prescription is highly regulated in Swiss prisons and BZD are administered in the health service office and consumed under supervision. This makes us confident that there was a high level of adherence to the prescribed type and dose of BZD. Another limitation is that the sample size was modest, resulting in a relatively small number of detained persons with punishable behaviors; consequently, punishable behaviors were analyzed as binary outcomes (present/absent). Further studies would benefit from inclusion of a larger number of detained persons. Due to small numbers of participants using the specific types of BZD, it was not possible to investigate effects of various types of BZD separately, resulting in a heterogeneous sample of individuals with BZD prescription. Types of BZD may have different relationships with aggression, so further studies should also investigate these differences, as well as the effects of dosage and duration of BZD use. There is also a question of the extent to which our findings could be generalized to prison populations in other countries and settings. Prospective studies of this topic in different countries, jurisdictions, and correctional settings are needed to confirm our preliminary findings.

### Conclusion

Detained persons are a vulnerable population with a high burden of psychiatric and general medical morbidity; they should receive appropriate, timely and evidence-based treatment without institutional barriers to treatment access. More specifically, we did not find support for the notions that BZD was associated with aggressive or disinhibited behavior or risk of substance abuse in detained persons. As with other pharmacological agents, BZD should be used carefully and cautiously in the prison setting, along with evidence-based psychological interventions.

## Supplementary Information

**Additional file 1: Table S1**. Types of benzodiazepines prescribed and guidelines for conversion into diazepam mg equivalents.

## Data Availability

The datasets used and/or analyzed during the current study are available from the corresponding author on reasonable request.
