# Supplementary material for: Relationship between benzodiazepine prescription, aggressive behavior, and behavioral disinhibition: a retrospective study in a Swiss prison
Source: Harm Reduct J. 2021 May 20;18:58. doi: 10.1186/s12954-021-00504-5 (PMC8139037; doi:10.1186/s12954-021-00504-5)
Supplement: Supplementary file 1 — Additional file 1: Table S1. Types of benzodiazepines prescribed and guidelines for conversion into diazepam mg equivalents. [file 12954_2021_504_MOESM1_ESM.docx]

Table S1. Types of benzodiazepines prescribed and guidelines for conversion into diazepam mg equivalents.

| Benzodiazepines | n (%) | Diazepam equivalents  (10 mg)* |
| --- | --- | --- |
| Alprazolam | 13 (3.9) | 1 |
| Bromazepam | 7 (2.1) | 6 |
| Clobazam | 1 (<0.1) | 20 |
| Clonazepam | 46 (13.9) | 2 |
| Diazepam | 112 (33.9) | 10 |
| Flurazepam | 7 (2.1) | 30 |
| Lorazepam | 76 (23.0) | 2 |
| Lormetazepam | 1 (<0.1) | 2 |
| Midazolam | 31 (9.4) | 7.5 |
| Nitrazepam | 5 (1.5) | 5 |
| Oxazepam | 90 (27.3) | 25 |
| Triazolam | 7 (2.1) | 0.5 |

* Diazepam mg equivalents were computed according to Caflisch [1]. For example, 1 mg of alprazolam equals 10 mg of diazepam.

1. Caflisch C: **Aequivalenztabelle**, Psychiatric University Hospital Zurich, Research Group on Substance Use disorders. 2018; Zurich, Switzerland, <https://praxis-suchtmedizin.ch/praxis-suchtmedizin/index.php/de/medikamente/benzodiabepine/aequivalenztabelle-bdz>
